# Antimicrobial resistance profile of *Escherichia coli* in drinking water from one health perspective in low and middle income countries

**DOI:** 10.3389/fpubh.2024.1440908

**Published:** 2024-12-03

**Authors:** Belay Desye, Temeselew Woldetsadik Mawugatie, Lakew Asmare, Yawkal Tsega, Dagnachew Melak, Abel Endawkie, Chala Daba

**Affiliations:** ^1^Department of Environmental Health College of Medicine and Health Sciences, Wollo University, Dessie, Ethiopia; ^2^Department of Economic, College of Management and Economics, Wollo University, Dessie, Ethiopia; ^3^Department of Epidemiology and Biostatistics, Institute of Public Health, College of Medicine and Health Sciences, University of Gondar, Gondar, Ethiopia; ^4^Department of Health System and Management, School of Public Health, College of Medicine and Health Sciences, Wollo University, Dessie, Ethiopia; ^5^National Center for Epidemiology and Population Health, The Australia National University, Dessie, Ethiopia; ^6^National Center for Epidemiology and Population Health, The Australia National University, Canberra, ACT, Australia

**Keywords:** antimicrobial resistance, *Escherichia coli*, drinking water, one health, low and middle income countries

## Abstract

**Introduction:**

Antimicrobial resistance is a major global public health concern, especially in low-resource settings. In low- and middle-income countries, the existing evidence about antimicrobial resistance in drinking water is inconsistence and not comprehensive. Therefore, this study aimed to estimate the pooled prevalence of antimicrobial resistance profiles of *Escherichia coli* from drinking water in low- and middle-income countries.

**Methods:**

This study was conducted using comprehensive literature searches using various databases such as PubMed, Scientific Direct, HINARI, and Google Scholar. Data extraction was performed using Microsoft Excel and exported to STATA 14/SE software for analysis. We used the Joanna Briggs Institute’s quality appraisal tool to ensure the quality of the included studies. A random effects model was employed to estimate the pooled prevalence. Publication bias was evaluated using funnel plots and Egger’s regression test. Subgroup and sensitivity analysis were also conducted in this study.

**Results:**

The study found that the pooled prevalence of *Escherichia coli* isolates in drinking water was 37.94% (95% CI: 26.73–49.13). The prevalence of multidrug resistance was 43.65% (95% CI: 31.15–56.15). Regarding specific antimicrobials, the pooled resistance levels of *Escherichia coli* were 54.65% (95% CI: 41.35–67.96) against contrimoxazole, followed by 48.64% (95% CI: −3.6–101) against amoxicillin and 48% (95% CI: −18.1–114.2) against cefuroxime.

**Conclusion:**

The findings indicated a significant prevalence of antimicrobial resistance of *Escherichia coli* isolated from drinking water and its multidrug resistance. To address this issue, it recommends focusing on improving basic hygiene and sanitation practices and enhancing water and wastewater treatment systems.

**Systematic review registration:**

Identifier CRD42024533592.

## 1 Introduction

Antimicrobial Resistance (AMR) occurs when microorganisms such as bacteria, viruses, fungi, and parasites no longer respond to antimicrobials, making infections difficult to treat (1). AMR occurs through genetic changes that occur naturally. This resistance can occur in humans, animals, and the environment (air, water, and soil). The drivers of AMR include misuse and overuse of antimicrobials, lack of access to clean Water, Sanitation and Hygiene (WASH), poor Infection Prevention and Control (IPC) practices, etc. (2, 3).

In our interconnected world, AMR has gained global attention as a significant public health challenge (4). The World Health Organization (WHO) has recognized AMR as one of the top ten global health threats (5). Additionally, AMR has been identified as a significant hurdle and one of the foremost challenges in attaining the Sustainable Development Goals (SDGs) (6–8). Consequently, addressing the escalating threat of AMR necessitates the implementation of a One-Health initiatives (9). One Health is a comprehensive and integrated approach that recognizes the interconnected nature and mutual dependence of the health of humans, animals, and the environment (10, 11).

In 2019, global reports indicated that 1.23 million deaths were attributed directly to AMR, and in addition, 4.95 million deaths were indirectly attributed to AMR (6). Disturbingly, projections suggest that by the year 2050, AMR could potentially lead to up to 10 million deaths per year worldwide (12). The impact of AMR is more severe in Low- and Middle-Income Countries (LMICs) where limited resources and inadequate implementation of WASH measures prevail. Insufficient sanitation facilities in these regions can contribute to water contamination and facilitate the transmission and spread of AMR (2, 13).

As per the United Nations, access to safe water and sanitation is recognized as a basic human right (14). However, AMR has emerged as a concerning contaminant in drinking water. Water plays a main role in the dissemination of AMR within the environment (15). Therefore, it is vital to establish a clean water supply system while improving the use of disinfectant chemicals or exploring effective options. This approach is important for improving water quality at the point of use and reducing the possible transmission of resistance bacteria through water sources (16).

Current treatment technologies used in water and wastewater treatment plants primarily focus on reducing physical and chemical contaminants, but they often provide limited removal of biological contaminants such as AMR (17). Moreover, there is often insufficient monitoring and assessment of AMR following water treatment (18). The water distribution system is recognized as a complex system, posing challenges for the inactivation and treatment of AMR. Despite the availability of advanced water treatment technologies like membrane filtration, activated carbon filtration, and advanced oxidation, these methods may not effectively treat AMR (19, 20). Enteric bacterial pathogens, particularly *Escherichia coli* (*E. coli*) isolates originating from drinking water, are a significant public health concern for humans (21, 22). The WHO has identified *E. coli* as a top priority pathogen and a main contributor to the AMR burden (23).

The identification of *E. coli* in drinking water samples in LMICs has gained increasing importance due to its potential risks to public health. Mahmud et al. (24) in Bangladesh reported that drinking water samples are sources of pathogenic *E.coli.* Many studies have revealed the occurrence of *E. coli* in drinking water samples in LMICs, with isolation rates ranging from 5.3% in Egypt (25) to 79.6% in Ghana (26). *E. coli* isolates from drinking water have shown resistance to antimicrobial agents such as cotrimoxazole, amoxicillin, ampicillin, and tetracycline (27–30). Multidrug resistance (MDR) has been observed among *E. coli* isolates from drinking water, with prevalence ranging from 19.7% in Peru (27) to 80% in Ethiopia (31).

The emergence and widespread prevalence of AMR present a significant challenge, especially in LMICs (32, 33). Although various countries worldwide have implemented antimicrobial stewardship programs to tackle the issue of AMR (17), and the WHO has launched a global AMR surveillance system (34). There are multiple studies conducted about AMR in health care settings, but there is limited evidence about the general environment, like drinking water, particularly in LMICs (35). Despite limited and inconsistent evidence, to the best of our literature search, no systematic review and meta-analysis has been conducted from a One Health perspective to examine the AMR profiles of *E. coli* isolates from drinking water in LMICs.

Therefore, the aim of this study is to estimate the overall prevalence of *E.coli* isolated from drinking water in LMICs. Through this review, the study aims to generate comprehensive evidence regarding the AMR profiles of *E. coli* isolates from drinking water in LMICs. This research will contribute to the One Health approach and support the attainment of the SDGs. Additionally, the findings of this study can inform decision-making processes and increase awareness among stakeholders and policymakers. Ultimately, the study has the potential to drive action and facilitate necessary interventions to tackle AMR in drinking water systems.

## 2 Methods

### 2.1 Study setting and protocol of registration

**Figure 1:**
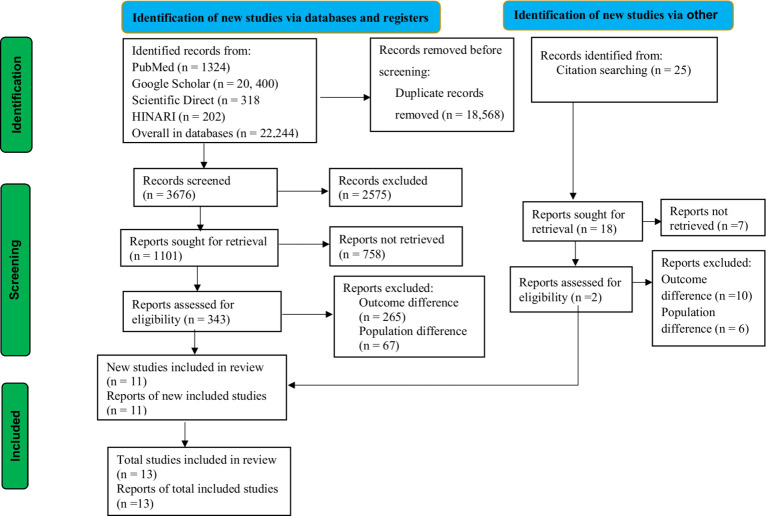
PRISMA flow diagram of this study, 2024.

The guidelines for updated Preferred Reporting Items for Systematic Reviews and Meta-Analysis (PRISMA) were used for this study (36) (Figure 1). The review protocol for this study was registered in the International Prospective Register of Systematic Reviews (PROSPERO) with the record id CRD42024533592. This study was conducted in LMICs, following the list of World Bank data (37).

### 2.2 Sources of information and search strategies

A systematic literature search was undertaken using a database of PubMed, Scientific Direct, HINARI, and Google Scholar. For example, for the PubMed search, key terms were used in combination with the Boolean operators “AND” and “OR.” Apart from conducting an electronic database search, additional articles were obtained by searching the gray literature through direct Google searches and by examining the bibliographies of the included articles.

### 2.3 Eligibility criteria

**Inclusion criteria:** this review included studies that fulfilled the following criteria.

- Population: articles conducted specifically on drinking water.
- Outcomes: articles reported the quantitative outcome of *E.coli* isolates (%), selected antimicrobial agents (%), and MDR of *E.coli* (%) in samples of drinking water.
- Study design: any observational studies (cross-sectional, case control, and cohort).
- Study setting: studies conducted in LMICs.
- Time frame: all studies reported from January, 02, 2000 to April, 30, 2024.
- Language of published studies: articles written in English.
- Publication issue: both published and unpublished studies.

***Exclusion criteria*:** studies that were not fully accessible despite three personal email contacts with the primary and corresponding authors, and studies that did not clearly show us the outcome interest of the study were excluded. Furthermore, certain types of research articles, including letters to editors, qualitative studies, systematic reviews, short communications, and commentaries, were not considered.

### 2.4 Study selection

Two investigators, BD and YT, conducted independent screenings of articles based on their titles, abstracts, and full texts to identify eligible articles. They followed pre-established criteria during this process. The screened studies were then combined by the two investigators, and any disagreements that arise during data abstraction and selection were resolved by discussions and the involvement of a third investigator (CD).

### 2.5 Data extraction and management

The format of data extraction for this study included author name, year of publication, country of the study, number of samples, number of *E.coli* isolates, % of *E.coli* isolates, % MDR of *E.coli* isolates, and risk of bias, these details were organized in a table format (Table 1). To collect articles and remove duplicate studies, Zotero Reference Manager was used. Additionally, the updated PRISMA checklist was employed to effectively summarize the study conditions (36) (Supplementary file 1).

**Table 1:** Summary of the included articles in AMR profiles of *Escherichia coli* isolates from drinking water in LMICs, 2024.

Author, publication year	Study country	No. of samples	No. of *Escherichia coli* isolates	*Escherichia coli* isolates (%)	Resistance of *Escherichia coli*	MDR of *Escherichia coli* (%)	Quality score (%)
Abera et al. (59)	Ethiopia	140	25	17.9	16.68	66.7	62.5
Ahmed et al. (28)	Ghana	524	115	21.9	67	58.2	75
Bonso et al. (60)	Ethiopia	100	68	68	23	33.8	75
Chen et al. (61)	China	404	200	49.5	49	24.5	75
Dhengesu et al. (29)	Ethiopia	75	20	26.7	-	-	75
Fakhr et al. (25)	Egypt	300	16	5.3	10	62.5	75
Hartinger et al. (62)	Peru	69	41	59.4	10.25	25	75
Kichana et al. (26)	Ghana	49	39	79.6	18.8	48.2	75
Larson et al. (27)	Peru	314	117	37.3	23	19.7	62.5
Odonkor et al. (30)	Ghana	110	23	20.9	6.4	27.8	75
Sahoo et al. (63)	India	417	151	36.2	-	-	75
Shakoor et al. (64)	Pakistan	100	35	35	-	-	62.5
Yenew et al. (31)	Ethiopia	60	24	40	19.2	80	62.5

-, not reported; *E.coli*, *Escherichia coli*; No., number, MDR, multidrug resistance which means resistance of antibiotics to three and more than three antibiotics.

### 2.6 Quality assessment of the studies

The quality appraisal tools of Joanna Briggs Institute (JBI) for analytical cross-sectional studies were used to assess the quality of the included studies (38). The quality of the articles was independently assessed by the two reviewers (BD and YT). Eight criteria were used to assess the quality of each article. The assessment options were categorized as yes, no, unclear, or not applicable. The risk of bias was classified as low (total score between 6 to 8), moderate (total score between 3 to 5), and high (total score between 0 to 2). Finally, articles scored more than 50% were considered in this study (39, 40), detailed assessment in Supplementary file 2.

### 2.7 Outcome of interest

This study has three main outcomes:

- The pooled prevalence of *E.coli* isolates in drinking water in LMICs, expressed as (%).
- The pooled prevalence of selected antimicrobial agents against *E.coli* in drinking water in LMICs, expressed as (%).
- The pooled prevalence of MDR of *E.coli* isolates in drinking water in LMICs, expressed as (%)

### 2.8 Statistical methods and data analysis

Microsoft Excel was used to extract data and transported to STATA version-14 for analysis. Index of heterogeneity (I2 statistics) was used to assess heterogeneity among the included articles, where values of 25–50%, 50–75%, and > 75% indicated low, moderate, and high heterogeneity, respectively (41). The metaprop command in STATA was used to estimate the pooled prevalence. Subgroup analysis to explore potential variations in the pooled prevalence in this study was conducted using study countries, sample size, and study year. In this study, the effect of each study on the estimated pooled results was assessed using sensitivity analysis. A funnel plot test and Egger’s regression test with a significance level of p < 0.05 as the cut point were used to ensure the presence of publication bias. To identify a possible heterogeneity source, a univariate meta-regression was employed. Finally, the findings of this study were presented using tables, figures, a forest plot, and descriptive text.

## 3 Results

### 3.1 Overview of search process

Using a database and other methods of search, a total of 22,244 studies were identified. After duplicate records were removed, 3,676 records were screened for this review. According to the records, only 1,101 reports were sought for retrieval. After being identified for retrieval, 343 reports were evaluated for eligibility. Following eligibility, a total of 332 studies were excluded due to differences in outcome interest and population differences. Ultimately, a total of 11 studies were included in this review from database sources. In addition to the database sources, 2 studies were included in this review from other sources. Finally, a total of 13 articles were included in this study, as presented in the PRISMA flowchart (Figure 1).

### 3.2 Characteristics of the eligible studies

All the included articles were cross-sectional studies. In this review, a total of 2,662 drinking water samples were included. Most of the studies were conducted in Ethiopia (*n* = 4) and Ghana (*n* = 3). The included studies were conducted between 2012 and 2023. In this study, the number of *E.coli* isolates was (*n* = 874), and the number of MDR of *E.coli* isolates was (*n* = 243.33). The *E.coli* isolates in this review ranged from 5.3% in Egypt (25) to 79.6% in Ghana (26), and the MDR of *E.coli* was found between 19.7% in Peru (27) and 80% in Ethiopia (31). All the included articles were categorized under moderate levels of risk of bias (Table 1).

### 3.3 The pooled prevalence of *Escherichia coli* isolates from drinking water in LMICs

**Figure 2:**
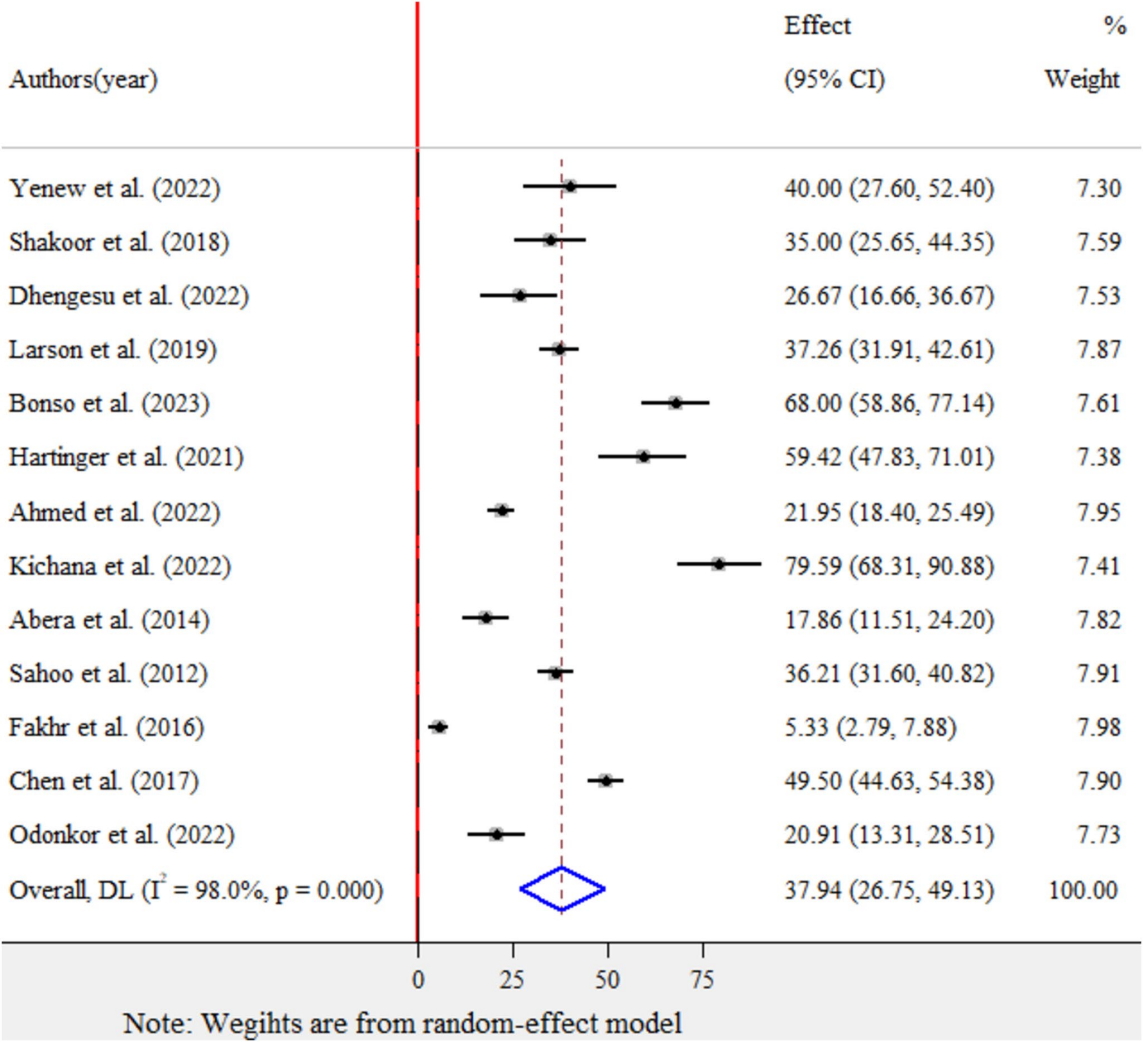
Forest plot for the pooled prevalence of *Escherichia coli* isolates from drinking water in LMICs, 2024.

The pooled prevalence of *E.coli* isolates using a random-effect model was estimated at 37.94% (95% CI: 26.75–49.13), with high heterogeneity (*I*^2^ = 98%, *p*-value < 0.001) (Figure 2). A sub-group analysis based on study countries, year of publication, and sample size was performed to assess the heterogeneity sources, which were presented in Table 2. The highest pooled prevalence of 49.5% (95% CI: 44.63–54.38) was observed in China, and the lowest pooled prevalence was estimated at 5.33% (95% CI: 2.79–7.88) in Egypt. Heterogeneity was highest among studies conducted in Ghana (I2 = 97.9%), followed by Ethiopia (*I*^2^ = 96.3%). Based on the year of publication, a study conducted in above 2018 was estimated at 43.87% (95% CI: 30–57.75) with (I^2^ = 96.4%, p-value < 0.001) and 28.72% (95% CI: 9.50–47.94) with (*I*^2^ = 98.8%, *p*-value < 0.001) for a study conducted in 2018 and below. In addition, a study conducted with a sample size of ≤ 200 found 43.22% (95% CI: 27.12–59.32) with (*I*^2^ = 95.9%, *p*-value < 0.001) and sample size of >200 found 29.97% (95% CI: 13.42–46.53) with (*I*^2^ = 98.9%, *p*-value < 0.001).

**Table 2:** Subgroup analysis of the pooled prevalence of *Escherichia coli* isolates from drinking water in LMICs, 2024.

Subgrouping criteria	Number of studies	Pooled prevalence (95% CI)	Heterogeneity	
			*I^2^*	*p*-value
Study country				
Ethiopia	4	38.04% (95% CI: 14.40–61.68)	96.3	<0.001
Pakistan	1	35% (95% CI: 25.65–43.25)	0.0	<0.001
Peru	2	47.72% (95% CI: 26.04–69.40)	91.4	0.001
Ghana	3	40.31% (95% CI: 12.60–68.01)	97.9	<0.001
India	1	36.21% (95% CI: 31.6–40.82)	0.0	<0.001
Egypt	1	5.33% (95% CI: 2.79–7.88)	0.0	<0.001
China	1	49.5% (95% CI: 44.63–54.38)	0.0	<0.001
Year of publication				
Above 2018	8	43.87% (95% CI: 30–56.75)	96.4	<0.001
2018 and below	5	28.72.% (95% CI: 9.50–47.94)	98.8	<0.001
Sample size				
≤200	8	43.22% (95% CI: 27.12–59.32)	95.9	<0.001
>200	5	29.97% (95% CI: 13.42–46.53)	98.9	<0.001

**Figure 3:**
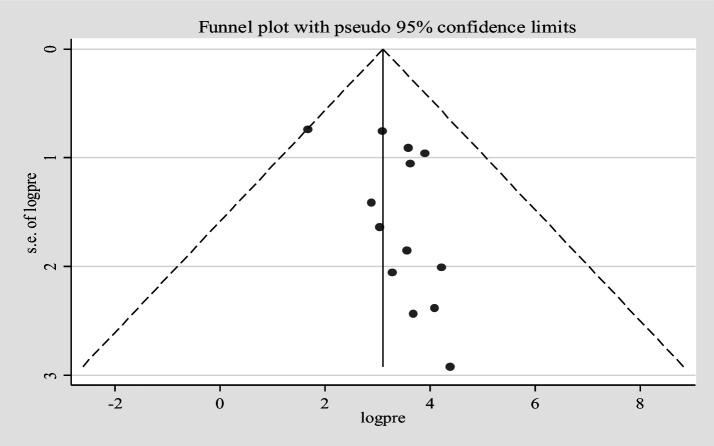
Funnel plot for *Escherichia coli* isolates from drinking water in LMICs, 2024.

Furthermore, univariate meta-regression was conducted using study country, sample size, and study year as factors to identify the source of heterogeneity. However, neither of them was found to be statistically significant as sources of heterogeneity (Supplementary file 3). A sensitivity analysis was also conducted to evaluate a single study effect. It was found that 37.94% (95%CI: 23.72–52.46) indicates a slightly broader confidence interval from the pooled prevalence of *E.coli* isolates, there is no a strong evidence for the effect of a single study (Supplementary file 4). In addition, the funnel plot showed that there was no evidence for publication bias, the included articles were symmetrically distributed (Figure 3). In addition to the funnel plot, the Egger-regression test confirmed that there is no publication bias for this study (*p*-value = 0.110).

### 3.4 Pooled prevalence resistance pattern of *Escherichia coli* isolates from drinking water in LMICs

Eleven antimicrobial agents were used to assess the resistance pattern of *E. coli*. The finding revealed a high pooled resistance level of *E.coli* was 54.65% (95% CI: 41.35–67.96) against contrimoxazole, 48.64% (95% CI: −3.6-101) against amoxicillin and 48% (95% CI: −18.1-114.2) against cefuroxime. However, a low pooled resistance level of *E.coli* was found at 15% (95% CI: 4.95–25.1) against for gentamicin and 15.73% (95% CI: 7.8–23.7) against ciprofloxacin (Table 3).

**Table 3:** Pooled prevalence of AMR patterns of *Escherichia coli* isolates from drinking water in LMIC, 2024.

Antimicrobial agents	Number of studies	Number of isolates	Number of resistance	Pooled prevalence of AMR (95% CI)	*I^2^* (%)	*p*-value
Ceftriaxone	4	185	114	33.9% (−13.4–81.2)	98.5	<0.001
Amoxicillin	3	255	85	48.64% (−3.6–101)	99.3	<0.001
Ciprofloxacin	9	532	96	15.73% (7.8–23.7)	87.8	
Trimethoprim-sulfamethoxazole	5	481	155	29.1% (11.7–46.5)	94.6	<0.001
Ampicillin	7	558	194	41.2% (19.1–63.3)	97.2	<0.001
Chloramphenicol	6	528	114	23.1%1 (14.8–31.4)	79.8	<0.001
Cefuroxime	3	255	118	48% (−18.1–114.2)	99.7	<0.001
Tetracycline	8	603	259	43.6% (32.5–54.8)	86.5	<0.001
Nalidixic acid	3	296	99	27.5% (−4.44–59.4)	97.7	<0.001
Gentamicin	5	204	28	15% (4.95–25.1)	78.2	<0.001
Cotrimoxazole	4	229	117	54.65% (41.4–68)	67.8	0.025

### 3.5 The pooled prevalence of MDR for *Escherichia coli* isolates from drinking water in LMICs

**Figure 4:**
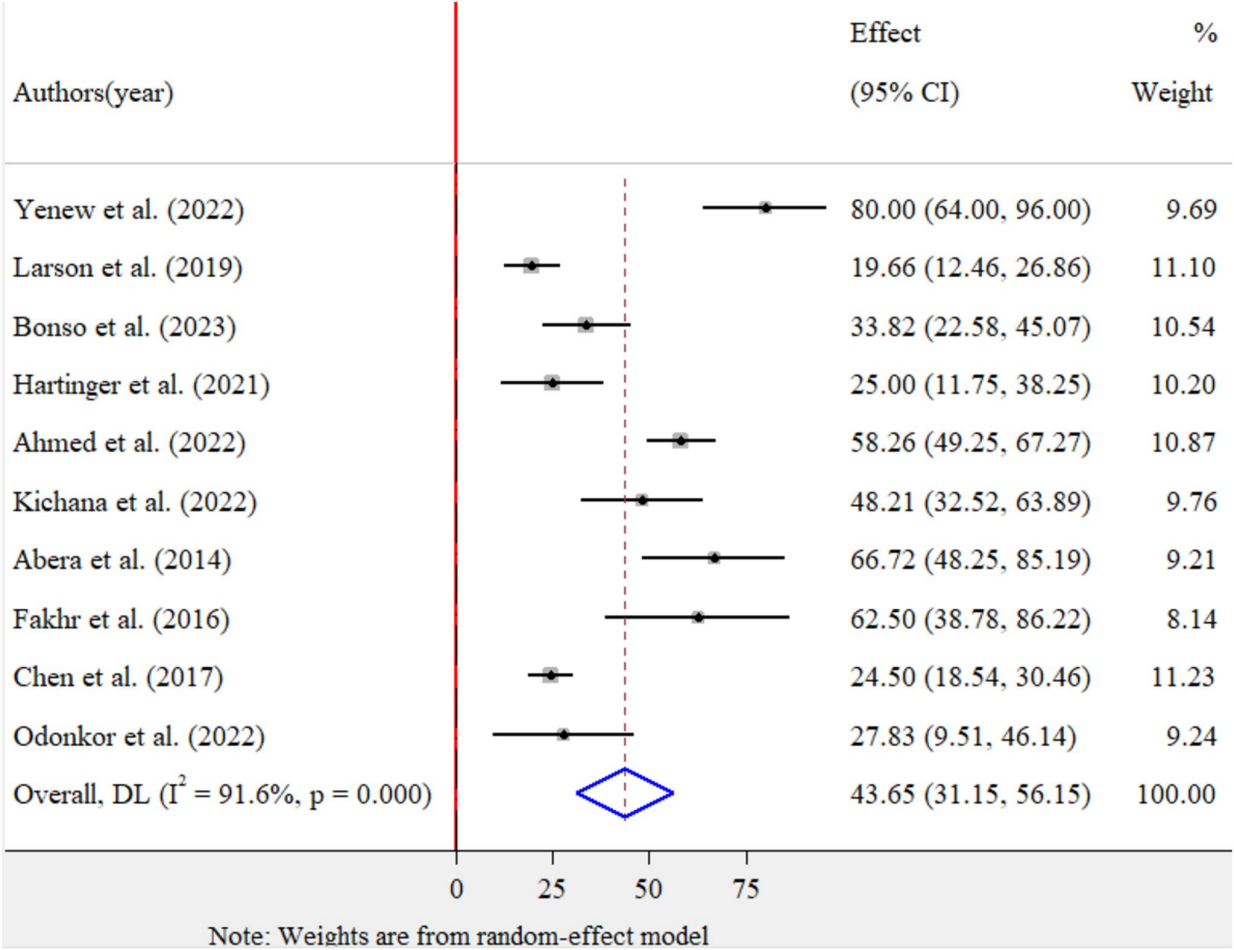
Forest plot for the pooled prevalence of MDR of *Escherichia coli* isolates from drinking water in LMICs, 2024.

**Figure 5:**
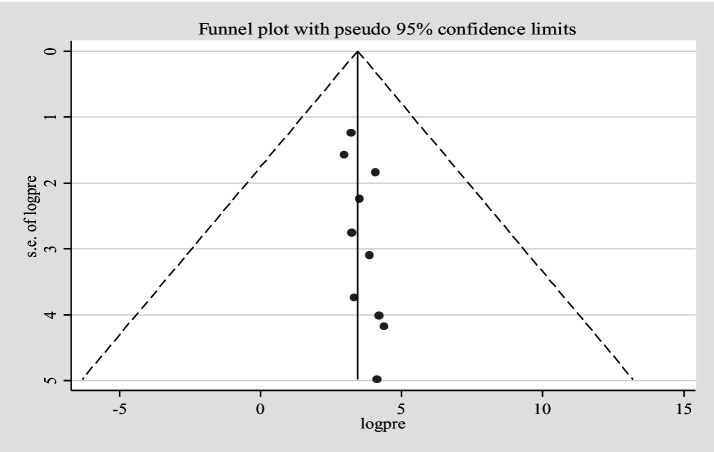
Funnel plot of MDR for *Escherichia coli* isolates from drinking water in LMICs, 2024.

The pooled prevalence of MDR for *E.coli* isolates using a random-effects model was estimated at 43.65% (95% CI: 31.15–56.15), with high heterogeneity (*I^2^* = 91.6%, *p-value* < 0.001) (Figure 4). A sub-group analysis based on study countries, year of publication, and sample size was performed to assess the heterogeneity sources, which were depicted in Table 4. The highest pooled prevalence of 62.50% (95% CI: 38.78–86.22) was observed in Egypt, and the lowest pooled prevalence was estimated 20.88% (95% CI: 14.55–27.20) in Peru. Heterogeneity was highest among studies conducted in Ethiopia (*I^2^* = 91.7%, *p-*value = <0.001), followed by Ghana (*I^2^* = 77.1%, *p-*value = 0.013). Based on the year of publication, a study conducted in above 2018 was estimated at 41.58% (95% CI: 25.58–57.78) with (*I^2^* = 92.3%, *p-*value < 0.001) and 50.06% (95% CI: 17.81–82.30) with (*I^2^* = 92.2%, *p-*value < 0.001) for a study conducted in 2018 and below. In addition, a study conducted with a sample size of ≤ 100 found 48.53% (*95%* CI: 32.66–64.40) with (*I^2^* = 85.7%, *p*-value < 0.001) and a studies conducted with sample size of >100 found 33.92% (95% CI: 13.10–54.74) with (*I^2^* = 95.6%, *p-*value < 0.001). Furthermore, univariate meta-regression was conducted using study country, sample size, and study year to identify the sources of factor for heterogeneity. However, neither of them was found to be statistically significant for sources of heterogeneity (Supplementary file 5). The findings of the sensitivity analysis indicated that it was 43.65% (95%CI: 28–60.69), only slightly broader in confidence interval from the pooled prevalence, that cannot assured the presence of the effect of a single study (Supplementary file 6). In addition, the funnel plot revealed that there was no evidence of publication bias, the included articles were symmetrically distributed (Figure 5). Moreover, the Egger- regression test confirmed that there is no publication bias for this study (*p*-value = 0.070).

**Table 4:** Subgroup analysis of the pooled prevalence of MDR of *Escherichia coli* isolates from drinking water in LMICs, 2024.

Subgroup criteria	Number of studies	Pooled prevalence (95% CI)	Heterogeneity	
			*I^2^* (%)	*p-*value
Study country				
Ethiopia	3	59.69% (95% CI: 29.47–89.90)	91.7	<0.001
Peru	2	20.88% (95% CI: 14.55–27.20)	0.0	0.488
Ghana	3	46.21% (95% CI: 29.19–63.22)	77.1	0.013
Egypt	1	62.50% (95% CI: 38.78–86.22)	0.0	<0.001
China	1	24.5% (95% CI: 18.54–30.46)	0.0	<0.001
Year of publication				
Above 2018	7	41.58% (95% CI: 25.58–57.58)	92.3	<0.001
2018 and below	3	50.06.% (95% CI: 17.81–82.30)	92.2	<0.001
Sample size				
≤100	7	48.53% (95% CI: 32.66–64.40)	85.7	<0.001
>100	3	33.92% (95% CI: 13.10–56.74)	95.9	<0.001

## 4 Discussion

AMR has emerged as a significant worldwide public health problem in the 21st century. It causes high rates of illness and death, particularly in LMICs (42). The presence of AMR exacerbates the high burden of bacterial infections in such areas, where access to adequate diagnostic tools is limited, antimicrobials are often misused or overused, and environmental conditions are poor (43, 44). The presence of AMR in drinking water increases the risk to human health. In this study, we estimate the prevalence and AMR patterns of *E.coli* isolates from drinking water in LMICs.

In this study, the pooled prevalence estimate of *E.coli* isolates from drinking water in LMICs was found to be 37.94% (95% CI: 26.75–49.13). This prevalence is lower than the 61.9% reported in a study of systematic reviews and meta-analysis conducted in Africa (45). The differences in prevalence could be attributed to variations in sample characteristics and the effectiveness of water treatment systems in place. The present findings might be explained by the lack of adequate water treatment and poor hygiene and sanitation conditions in those resource-constrained settings (44). However, WHO recommends drinking water be free from *E.coli* (46). Therefore, it is crucial to strengthen AMR stewardship efforts and improve hygiene and sanitation conditions to address this issue effectively.

In this study, *E. coli* demonstrated a high level of resistance to several antimicrobials. The highest resistance rates were observed for cotrimoxazole (54.65%), amoxicillin (48.64%), cefuroxime (48%), tetracycline (43.6%), and ampicillin (41.2%). On the other hand, the lowest resistance rates were found for gentamicin (15%), ciprofloxacin (15.73%), and chloramphenicol (23.1%). The current result is supported by many studies showing that *E.coli* is resistant to many antimicrobials. For example, studies have shown *E.coli* resistance rates of 69.4 and 77% for ampicillin (45, 47). Another study documented the resistance of *E. coli* to amoxicillin (24.5%), ampicillin (23.5%), chloramphenicol (12.3%), and trimethoprim-sulfamethoxazole (22.5%) (42). In addition, studies reported lower resistance rates for *E.coli* rates for ciprofloxacin, ranging from 3 to 13.1% (45, 47). The observed variations in the reported resistance rates could be due to geographical locations and the nature of the study samples. Different countries may have varying levels of antimicrobial use, healthcare practices, surveillance, and sanitation systems, which can influence the resistance level of *E.coli* to each antimicrobial. Additionally, the condition of the samples analyzed, the source of the sample, and the setting conditions can also contribute to the differences in resistance rates (45, 47).

Overuse of these antibiotics and a lack of safe disposal may lead to resistance by promoting resistance development (48). For instance, cefuroxime is one of the major resistances against *E.coli* in this study, it is effective against Entrobacteriaceae bacteria, and it is a second-generation cephalosporin antibiotic (49). It is also used for the treatment of urinary tract infections (50). Therefore, the results of the resistance of *E.coli* in this study may be due to inappropriate treatment of human and animal wastes and their disposal in the environment. Hence, these untreated water sources contribute to AMR dissemination (28).

In this study, the pooled prevalence of MDR in *E. coli* was found to be 43.65%. This prevalence is lower than the 50.7% reported in a study conducted on water samples in Africa (45). The present finding is supported by earlier studies, which have highlighted the continued significance of *E. coli* resistance (42, 51). Additionally, in humans, animals and the environment, there have been reports for a high prevalence of AMR (47, 52, 53). Mostly, the present water treatment methods are unable to treat AMR adequately (48). Thus, improving basic hygiene and sanitation and advancing the treatment system will reduce the spread of resistant organisms (44).

LMICs are disproportionally affected by AMR due to the high infectious disease burden and poor antimicrobial use control and regulation (54). The sources of resistance can be environmental sources like sewerage systems, abattoirs, and waste from healthcare facilities that cannot be treated adequately and can contaminate drinking water sources (55). Although treatment systems for water and wastewater have been effective in reducing antimicrobial levels, findings revealed that antimicrobials are still present in drinking water (56, 57). This is a matter of concern because the existence of antibiotics in the environment can exert a strong selective pressure, promoting the acquisition and spread of resistance mechanisms among bacteria (58). Given these implications, it is highly recommended to advance and improve treatment systems to safeguard against the development and dissemination of AMR. By implementing more advanced treatment processes, it can minimize the existence of antimicrobials in sources of water.

### 4.1 Limitation of the study

This study was limited to publications in the English language and did not consider studies in other languages. In addition, this study focused exclusively on *E.coli* isolates and their resistance, neglecting the importance of multiple other enteric pathogens that have significant implications for public health. Moreover, the study did not identify the factors associated with *E.coli* resistance in drinking water.

## 5 Conclusion

AMR has emerged as a critical worldwide public health issue, particularly in LMICs. This study highlights *E.coli* isolates and their resistance to drinking water in LMICs were prevalent. The study revealed that *E. coli* resistance to various antimicrobial agents, with high resistance observed for contrimoxazole, amoxicillin, cefuroxime, and tetracycline. To tackle this concerning issue, it is essential to improve basic hygiene and sanitation practices. Additionally, advancing and upgrading water and wastewater treatment systems is essential to minimize the spread of resistant organisms. Taking a One-Health approach, it is recommended that concerned bodies and international organizations collaborate to mitigate health risks and minimize the environmental impact of AMR. For future researchers, it is advisable to conduct comprehensive investigations of other enteric pathogens in drinking water. Furthermore, identifying the drivers or factors contributing to AMR in drinking water would provide valuable insights for developing targeted interventions and strategies. By taking these actions, researchers and policymakers can work together to tackle the critical challenge of AMR in drinking water, particularly in LMICs where the burden is most severe.

## Data Availability

The raw data supporting the conclusions of this article will be made available by the authors, without undue reservation.
