# Supplementary material for: Antimicrobial resistance profile of Escherichia coli in drinking water from one health perspective in low and middle income countries
Source: Front Public Health. 2024 Dec 3;12:1440908. doi: 10.3389/fpubh.2024.1440908 (PMC11653505; doi:10.3389/fpubh.2024.1440908)
Supplement: Supplementary file 1 [file Table_1.DOCX]

**Supplementary File 6: Sensitivity analysis of resistance of *E.coli* isolates from drinking water in LMIC, 2024.**
