# Supplementary material for: Antimicrobial resistance profile of Escherichia coli in drinking water from one health perspective in low and middle income countries
Source: Front Public Health. 2024 Dec 3;12:1440908. doi: 10.3389/fpubh.2024.1440908 (PMC11653505; doi:10.3389/fpubh.2024.1440908)
Supplement: Supplementary file 3 [file Table_3.DOCX]

**Supplementary File 3: Univariate Meta-regression for the pooled prevalence of *E.coli* isolates from drinking water in LMICs, 2024.**

| **Variable** | **Coefficient** | ***p-value*** | **95%CI** | |
| --- | --- | --- | --- | --- |
| Study year | -0.068 | 0.736 | -0.514 | 0.377 |
| Sample size | -0.038 | 0.895 | -0.67 | 0.599 |
| Study country | -0.056 | 0.943 | -1.77 | 1.658 |
