# Supplementary material for: Antimicrobial resistance profile of Escherichia coli in drinking water from one health perspective in low and middle income countries
Source: Front Public Health. 2024 Dec 3;12:1440908. doi: 10.3389/fpubh.2024.1440908 (PMC11653505; doi:10.3389/fpubh.2024.1440908)
Supplement: Supplementary file 5 [file Table_5.DOCX]

**Supplementary File 5. Univariate Meta-regression for the pooled prevalence of MDR for *E.coli* isolates from drinking water in LMICs, 2024.**

| **Variable** | **Coefficient** | ***p-value*** | **95%CI** | |
| --- | --- | --- | --- | --- |
| Study year | 0.005 | 0.993 | -1.45 | 1.46 |
| Sample size | -0.033 | 0.946 | -1.18 | 1.12 |
| Study country | -0.033 | 0.984 | -3.84 | 3.78 |
