# Supplementary material for: Antimicrobial resistance profile of Escherichia coli in drinking water from one health perspective in low and middle income countries
Source: Front Public Health. 2024 Dec 3;12:1440908. doi: 10.3389/fpubh.2024.1440908 (PMC11653505; doi:10.3389/fpubh.2024.1440908)
Supplement: Supplementary file 6 [file Table_6.DOCX]

**Results of JBI Quality Assessment**

| Studies | Clear eligibility criteria | Description of study subject and study setting | Valid and reliable method to measure the exposure | Standard criteria used for measurement of the condition | Identification of confounding factors | Develop of strategies to deal with confounding factors | Valid and reliable method to measured outcomes | Appropriate statistical analysis | Total score out of 8 | Quality score (%) |
| --- | --- | --- | --- | --- | --- | --- | --- | --- | --- | --- |
| Yenew et al. (2022) | Yes | Yes | Yes | Yes | N/A | N/A | Yes | No | 5 | 62.5 |
| Shakoor et al. (2018) | Yes | Yes | Yes | Yes | N/A | N/A | Yes | Yes | 6 | 75 |
| Dhengesu et al. (2022) | Yes | Yes | Yes | Yes | N/A | N/A | Yes | Yes | 6 | 75 |
| Larson et al. (2019) | Yes | Yes | Yes | Yes | N/A | N/A | Yes | Yes | 6 | 75 |
| Bonso et al. (2023) | Yes | Yes | Yes | Yes | N/A | N/A | Yes | Yes | 6 | 75 |
| Hartinger et al. (2021) | Yes | Yes | Yes | Yes | N/A | N/A | Yes | Yes | 6 | 75 |
| Ahmed et al. (2022) | Yes | Yes | Yes | Yes | N/A | N/A | Yes | Yes | 6 | 75 |
| Kichana et al. (2022) | Yes | Yes | Yes | Yes | N/A | N/A | Yes | Yes | 6 | 75 |
| Abera et al. (2014) | No | Yes | Yes | Yes | N/A | N/A | Yes | Yes | 5 | 62.5 |
| Sahoo et al. (2012) | Yes | Yes | Yes | Yes | N/A | N/A | Yes | Yes | 6 | 75 |
| Fakhr et al. (2016) | Yes | Yes | Yes | Yes | N/A | N/A | Yes | Yes | 6 | 75 |
| Chen et al. (2017) | No | Yes | Yes | Yes | N/A | N/A | Yes | Yes | 5 | 62.5 |
| Odonkor et al. (2022) | No | Yes | Yes | Yes | N/A | N/A | Yes | Yes | 5 | 62.5 |
